# Record of thanatology and cannibalism in drills (*Mandrillus leucophaeus*)

**DOI:** 10.1007/s10329-023-01075-8

**Published:** 2023-06-27

**Authors:** Grazia Casetta, Andrea Paolo Nolfo, Elisabetta Palagi

**Affiliations:** 1 Unit of Ethology, Department of Biology, University of Pisa, Via A. Volta 6, 56126 Pisa, Italy; 2 Natural History Museum, University of Pisa, Via Roma 79, 56011 Pisa, Calci Italy

**Keywords:** Post-mortem care, Death-related behaviors, Infant gaze engagement, Filial cannibalism, Old World monkeys

## Abstract

Comparative thanatology includes the scientific study of death in non-human animals, which involves emotional, social, and exploratory responses of individuals and groups towards corpses. Stillborn babies and dead infants often elicit maternal and alloparental care that can persist for days, weeks, or even months, especially in primates. After this period, cannibalistic acts can occur not only by groupmates but also by the mother. Such cannibalism has been reported both in captive and wild primate groups, suggesting that the phenomenon is evolutionary adaptive. Here, we report a case in drills (*Mandrillus leucophaeus*), a largely unstudied monkey species. We collected data from the birth to the death of the infant on maternal and alloparental care towards the newborn across three phases: pre-mortem, post-mortem, and post-mortem cannibalism. The mother maintained high levels of grooming after the infant’s death. Both the mother and other group members interacted with the dead baby by trying to engage its gaze. Two days after the death, the mother started to eat the corpse until it was almost completely consumed; there was no sharing with other group members. Although we cannot draw firm conclusions about potential benefits of the mother’s behavior, this observation on drills adds a piece to the puzzle of thanatological behaviors and cannibalism in primates.

**Supplementary Information:**

The online version contains supplementary material available at 10.1007/s10329-023-01075-8.

## Introduction

The scientific study of death-related phenomena in non-human animals falls within the domain of comparative thanatology, which investigates emotional, social, and exploratory responses of individuals towards the corpse (Anderson 2017). Although several death-related behaviors (e.g., continuing maternal care, grief, vigils, visitations, guarding) were for long considered as exclusively human (Bercovitch 2020; Gonçalves and Biro 2018), more recently they have been related to the cooperative, succoring, and protective propensities in social mammals (de Waal and Preston 2017; Pérez-Manrique and Gomila 2018). Individuals may actively support dying or dead conspecifics by pulling, shaking, or lifting them (chimpanzees, *Pan troglodytes*, Anderson 2011; elephants, *Loxodonta africana*, Douglas-Hamilton et al. 2006; dolphins, *Delphinus capensis*, Park et al. 2013). A group of rhesus macaques (*Macaca mulatta*) was observed repeatedly performing such vigorous actions on an individual that had temporarily lost consciousness after being electrocuted on railway tracks (De Marco et al. 2022).

Stillborn babies and dead infants often elicit maternal care that can persist for days, weeks, or even months in non-human primates (see Anderson 2011; Watson and Matsuzawa 2018; Gonçalves and Carvalho 2019), but similar responses have also been observed in other mammals such as elephants (Goldenberg and Wittemyer 2020), and cetaceans and other aquatic mammals (Reggente et al. 2016). These behaviors, which can be associated with the hormonal state of the mother and/or her emotional attachment to the infant (Biro et al. 2010), possibly reflect a lack of understanding by the mother about the death of the baby (Hrdy 1979). This hypothesis could explain why the nulliparous or otherwise inexperienced mothers may be more likely to persist in caring for or carrying dead infants (Biro et al. 2010; Warren and Williamson 2004), although this is not a universal pattern in primates (Sugiyama et al. 2009; Das et al. 2018). Fernández-Fueyo et al. (2021) identified some possible predictors of the occurrence of infant corpse-carrying by mothers. Their phylogenetic regression approach revealed that the phenomenon occurs when few contextual or physical hints about the death of the infant are available to the mother; this supports the “death detection hypothesis” regarding possible awareness-of-death capacity in primates (Fernández-Fueyo et al. 2021).

Maternal care of infant corpses is the most frequently documented response to death by monkeys and apes in both natural and captive settings (Anderson 2011; King 2013; Sugiyama et al. 2009). Most females abandon the decomposing body of their infant within 1 week of the death (Fashing et al. 2011; Sugiyama et al. 2009), but some may carry the corpse for periods ranging from 2 weeks to 2 months (Biro et al. 2010; Fashing et al. 2011; Sharma et al. 2011; Sugiyama et al. 2009; Watson and Matsuzawa 2018). In some cases, mothers reportedly ate the corpse, consuming it completely (Fedurek et al. 2020).

Cannibalism, which includes “killing and consuming a conspecific in part or as a whole” (Fouilloux et al. 2019, p. 1295), may seem an evolutionary paradox, but benefits include providing animals with energy and nutrients, or with a reduced number of potential competitors (Bose 2022). In primates, cannibalism can follow infanticide (Culot et al. 2011; Fedurek et al. 2020) carried out by members of either sex, unrelated or related to the infant, and even by parents (Hrdy 1979). Several hypotheses have been proposed to explain cannibalism, linked to reduced competition for limited resources (Townsend et al. 2007) and increased opportunities for males to sire offspring (Hrdy 1979; van Schaik 2000). In primates, however, infanticide and cannibalism do not necessarily co-occur. For example, in chimpanzees, cannibalism has been observed following the natural death of an infant (Fedurek et al. 2020), and although infanticide victims are often at least partly consumed by the killer and other group members (marmosets, Bezerra et al. 2007; tamarins, Culot et al. 2011; orangutans, Dellatore et al. 2009; chimpanzees, Watts and Mitani 2000; snub-nosed monkeys, Xiang and Grueter 2007), this does not always happen (e.g., rhesus monkeys, Tian et al. 2016).

Although reports of killing and consumption of infants by mothers are rare in free-ranging primates (Watson and Matsuzawa 2018), filial infanticide may occur when the newborn suffers some impairments that prevent normal development (Lukas and Huchard 2019). However, partial consumption of the infant’s body without evidence of prior killing by a conspecific may occur, i.e., “passive cannibalism” (Anderson 2018). Filial cannibalism has been interpreted by some scholars as arising from stressful conditions (see Culot et al. 2011; Dellatore et al. 2009) and by others as a possible feature of the natural behavioral repertoire (e.g., Tonkean macaques, De Marco et al. 2018; bonobos, Tokuyama et al. 2017). As maternal cannibalism can provide nutritional benefit to the mother (Bose 2022) and can be associated with maternal infanticide (e.g., rodents, Elwood 1991), it has been hypothesized that the two phenomena may co-occur in cases of low infant survival probability. Finally, cannibalism can accelerate the mother’s detachment from her offspring by stopping caregiving activities, further enabling resumption of reproductive activities (sensu Klug and Bonsall 2007).

Here, we describe a case of brief maternal care after the death of a baby, followed by a cannibalistic behavior by the mother in *Mandrillus leucophaeus*, a little-studied and endangered primate species (Gadsby et al. 1994; Marty et al. 2009). The mother involved was a multiparous female (Kumasi) with previous experience of the death of one of her infants.

## Methods

Drills (*Mandrillus leucophaeus*) are semi-terrestrial, highly sexually dimorphic, forest-dwelling monkeys (Marty et al. 2009). They live in groups of 20–40 individuals, which generally contain a single reproductive male. The social organization seems to be flexible with groups that sometimes may split into sub-groups or fuse to form super-groups ranging from 100 to 300 individuals (Abernethy et al. 2002). Similar to mandrills, drills are seasonal breeders, with females reaching sexual maturity at about 3.5 years and gestation lasting from 168–179 days (Dezeure 2021).

The study group, all members of which were captive born, lived in the Safari Park Dvůr Králové, Czech Republic, in an outdoor area of 1575 m^2^. They also had free access to an indoor area of 50 m^2^, which was enriched with wooden structures, perches, and ropes and in which food (commercial primate pellets, fresh fruit, and vegetables) was distributed around feeding time (08:00 h–12:00 h). Water was available ad libitum. All subjects (Table 1) were individually recognized based on distinctive features such as sex, size, permanent scars, and missing fur patches.

**Table 1:** Subjects of the study and presence/absence of interaction with the newborn

Name	Age	Sex	Behavior towards the newborn			
			Grooming/inspecting		Contact/embracing	
			Pre-mortem	Post-mortem	Pre-mortem	Post-mortem
Atu	Infant	Male	Yes	No	Yes	Yes
Chepo	Adult	Male	Yes	No	Yes	Yes
Efuru	Adult	Female	Yes	Yes	Yes	Yes
Kali	Infant	Female	Yes	No	Yes	Yes
Kara	Juvenile	Female	Yes	No	Yes	Yes
Kebale	Adult	Female	No	No	Yes	Yes
Kumasi the mother	Adult	Female	Yes	Yes	Yes	Yes
Kwai	Juvenile	Female	Yes	No	Yes	Yes
Mambilla	Juvenile	Male	No	No	No	Yes
Mumba	Juvenile	Male	No	No	Yes	No
Ndolo	Sub-adult	Male	No	Yes	Yes	Yes
Obudu	Sub-adult	Male	No	No	Yes	Yes
Ricardo	Adult	Male	No	No	Yes	No

A study on the drills’ social behavior took place from August to October 2020. During this period, we conducted daily observations lasting about 8 h, divided into two sessions (09.00 h–12.30 h; 15.00–18.30 h). Video recordings were made with the aid of two video cameras (Canon EOS 110D; Full HD Panasonic Lumix DC-FZ82), which allowed us to closely film the focal mother with her infant. As the frame covered about 4 m around the dyad, we also recorded behaviors that other group members directed towards the mother–infant pair, and used the videos to establish with precision when each drill might be able to interact with the dyad. A total of about 11 h of video was collected, coinciding with when the drills were visible to the observers. All behavioral patterns recorded are shown in Table S1.

Two observers (authors APN and GC) independently analyzed 2 h of videos to assess inter-observer agreement (Kaufman and Rosenthal 2009). For each behavior listed in Table S1, Cohen’s *κ* was never below 0.85; for specific behavioral categories analyzed, Cohen’s *κ* was: grooming/visual 88.4%, tactile 87.8%, olfactory inspecting 85.3% (pooling all actions involving multisensory exploration of the infant) and body contact/embracing 89.1%. To analyze time spent by grooming/inspecting and in body contact/embracing the newborn by the mother, we applied a Chi-square test. The exact Friedman test was employed to assess whether grooming/inspecting and body contact/embracing by the group members towards the mother–newborn dyad differed across the three observation phases (pre-mortem, post-mortem, and post-mortem cannibalism); in the case of a significant difference, we followed up with the Dunnett's multiple comparison test (post hoc test with Bonferroni correction). Statistical analyses were performed using SPSS 20.0.

## Results

### Descriptive results

On August 24, 2020, a baby was born, and on September 2nd the observers realized that it was dead (pre-mortem phase). The newborn had appeared not to be in good health, as the mother had to actively hold him; he did not grasp her fur or reach the nipple. Although during the first 6 days no aggression towards the baby was observed, we cannot exclude that infanticide took place. The mother and other group members interacted with the corpse for 2 days (post-mortem phase) (Table 1). On September 3rd, the mother started to eat the corpse until it was almost completely consumed (post-mortem, cannibalism phase). The following day, park staff removed the remaining parts of the corpse (skull, limbs, and tail) from the enclosure. Below, we describe the most salient events following the infant’s death.

September 2nd—post-mortem phase (video 8:32–11:40)—At 8.42 we observed Kumasi holding and dragging the baby’s corpse while avoiding Chepo (the dominant male) (Video 1). She grooms it (Video 2) while shunning another adult female, Efuru, who is interested in it. At 9:30 two young adult males (Ndolo and Obudu) try to engage the dead baby’s gaze (Video 3a, b); Chepo prevents Ndolo from approaching Kumasi and the dead infant (Video 4). At 9:57, Efuru grooms the dead infant (Video 5). At 11:26, Kumasi closely sniffs the corpse, while Ndolo approaches Kumasi, touches the baby, and makes nose-to-nose contact with the mother (Video 6). At 11:33, Obudu approaches Kumasi, makes nose-to-nose contact with her, and tries to engage the baby’s gaze. When Obudu touches the baby, the mother moves away, dragging it. Obudu aggressively pushes the mother twice, and Chepo approaches Kumasi and tries to engage the dead infant’s gaze. Some females try to make nose-to-nose contact with the mother while touching and inspecting the corpse. (video 17:06–17:20)—Kumasi holds and licks the baby (17:06–17:11). At 17.17, Atu (an infant male) tries to steal it but Kumasi protects it; then she grooms and explores its head and limbs, until 17:20.

September 3rd—post-mortem phase (video 10:20–12:03)—Kumasi holds the baby while feeding (10:20–10:53). At 11:00, Kumasi starts a reciprocal grooming session with Kwai (a juvenile female), with Ndolo nearby, sniffing the baby. At 11:02, Kumasi starts grooming corpse. At 11:15, Atu and Ricardo, beside Kumasi, and look at and sniff the baby. At 11:23, Kumasi sniffs and grooms the corpse while protecting it from approaches of the males.

(Video 17:03–17:54)—At 17:09, Kumasi attacks Atu twice, and after which Atu presents her rear to Kumasi. At 17:10, Kumasi starts grooming her baby. At 17:23, she jumps onto a branch and, for the first time, loses her grip on the corpse, which falls down to the ground (Video 7). At 17:24, Obudu throws the corpse away and drags it while spinning around (Video 8). Obudu and Kumasi alternate in dragging around and spinning the corpse. Post-mortem cannibalism phase—At 17:35, Kumasi starts eating the head of the baby; this lasts 1 min 30 s (Video 9). At 17:36, Obudu approaches the corpse and sniffs it; Kumasi avoids him. Obudu sits near Kumasi, while she continues feeding on the corpse (head and abdomen) for 5 min 45 s. At 17:39, Obudu tries to steal the corpse, but Kumasi fends him off. At 17:41, Kumasi continues feeding on the abdomen for 6 min 30 s. In the next 4 min, Obudu tries to sniff the corpse but Kumasi avoids him. At 17:46, Kumasi runs away from Kara (a juvenile female) with the corpse. At 17:47, Kara sniffs the corpse for 2 min 16 s. Kumasi feeds on the corpse for 4 min and 30 s.

September 4th day 3—Post-mortem cannibalism phase (video 10:03–11:16)—At 10:03, Kumasi feeds on the corpse for 17 min. (Video 17:00–17:30)—At 17:24, Kumasi feeds on the corpse for 4 min 25 s.

### Statistical results

**Fig. 1:**
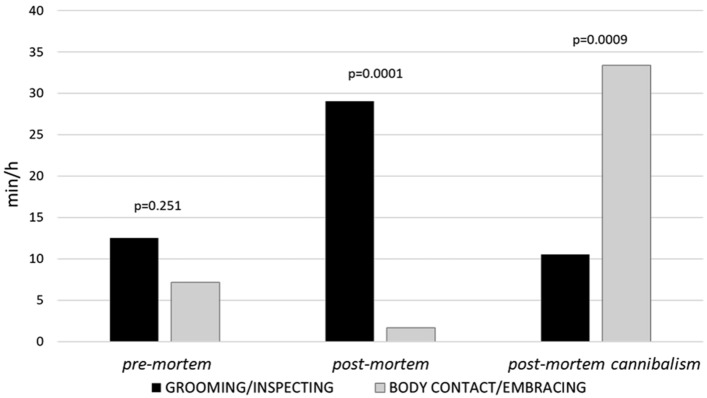
Bar graphs showing the time spent (minutes per hour of observation) by the mother in grooming/inspecting (*black bars*) and contact/embracing (*grey bars*) the baby in *pre-mortem* (August 24th–September 1st), *post-mortem* (September 2nd–3rd) and *post-mortem cannibalism* (September 3rd) phases

For the three phases, we calculated the amount of minutes per each hour animals spent in grooming/inspecting (GR_IN) and contact/embracing (BC_EM) by the mother towards the newborn. The time spent in GR_IN and BC_EM did not differ during pre-mortem phase (*χ*^2^ = 1.32, *df* = 1, *p* = 0.251). During the *death* phase the mother showed more GR_IN than BC_EM (*χ*^2^ = 24.00, *df* = 1, *p* = 0.001); conversely, during the *post-mortem cannibalism* phase the mother’s amount of time spent in BC_EM was significantly greater than GR_IN (*χ*^2^ = 11.00, *df* = 1, *p* = 0.009) (Fig. 1).

**Fig. 2:**
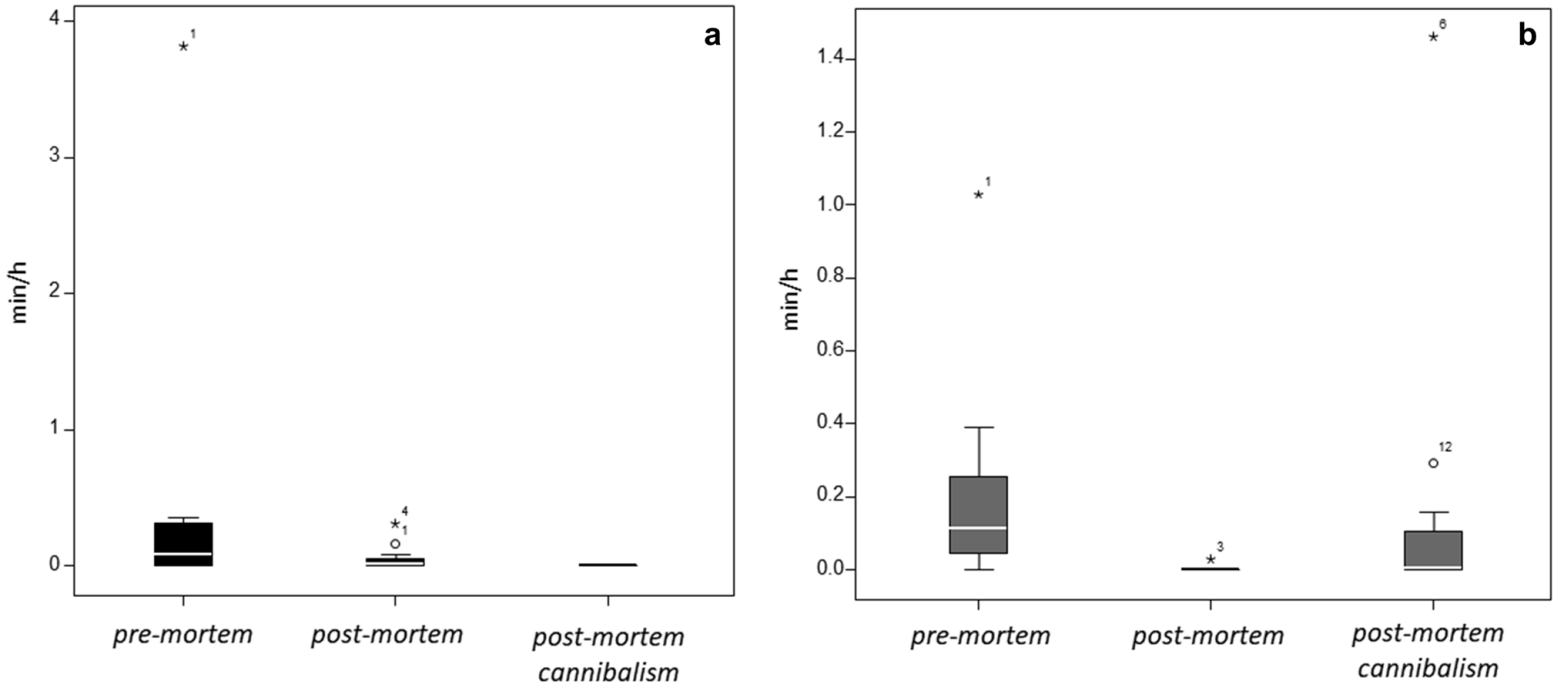
Boxplots showing the time spent (minutes per hour of observation) by group members in grooming/inspecting (**a**) and contact/embracing (**b**) the mother in pre-mortem (August 24th–September 1st), post-mortem (September 2nd–3rd) and post-mortem cannibalism (September 3rd) phases

The amount of GR_IN and BC_EM by other group members did not differ across the three phases (exact Friedman test: *χ*^2^_GR_IN_ = 2.13, *N* = 12, *p* = 0.371; *χ*^2^_BC_EM_ = 6.25, *N* = 12, *p* = 0.059). However, the frequency of GR_IN directed by other group members to the mother differed across the three phases (*χ*^2^_GR_IN_ = 7.00, *N* = 12, *p* = 0.028). GR_IN was slightly though non-significantly more frequent during the pre-mortem compared to the post-mortem phase (Dunnett's test *p* = 0.057); no differences were found between the other conditions (Fig. 2a).

The frequency of BC_EM by other group members toward the mother differed across the three phases (*χ*^2^_BC_EM_ = 14.80, *N* = 12, *p* = 0.0001), with BC_EM significantly more frequent during the pre-mortem compared to the post-mortem cannibalism phase (Dunnett's test *p* = 0.003) being the only significant difference (Fig. 2b).

## Discussion

This is the first report of post-mortem carrying and cannibalism of an infant in a captive group of drills. The infant was born during the observation period and survived for 8 days. We have no precise information on the cause of the death. In the pre-mortem phase, the mother and other group members tried to engage the gaze of the baby, who sometimes appeared to respond suggesting a degree of social activity of the infant. In the pre-mortem and post-mortem phases, all group members showed heightened interest in the mother, with the dominant male actively defending the corpse, but this behavior disappeared in the period involving cannibalism (Fig. 2). Preventing younger/lower-ranking groupmates from closely examine the corpse, a behavior described as ‘guarding’, has been reported in a few primate species (Gonçalves and Carvalho 2019). Pérez-Manrique and Gomila (2018) proposed that such ‘protective’ behavior shared mechanisms with sympathetic concern and empathetic targeted helping phenomena.

After her baby’s death, the mother continued to take care of him for 2 days. Her increased tactile and visual exploration (measured by grooming and inspecting) of the corpse may indicate attempts to get a reaction from the baby. Interestingly, other group members also tried to engage the gaze of the dead infant. This observation recalls reports that both monkeys and apes often inspect the face and/or eyes of recently deceased individuals, possibly to detect the absence of eye movements (De Marco et al. 2018). The persistent absence of gaze engagement may be a cue that “something is wrong” with the dead individual.

In many monkey species, juveniles and adult females are strongly attracted by dead infants with adult males showing less or no interest (De Marco et al. 2022). Yet, in chimpanzees (Cronin et al. 2011) and common marmosets (Thompson et al. 2020) adult males may also show some form of interest towards infant corpses. As described in other species, corpse-directed behaviors shown by the adult male and other drills in our study group included grooming, gazing, sniffing, touching, and dragging. Some authors suggest that these actions may serve to test the corpse’s responsiveness (Anderson et al. 2010; De Marco et al. 2020; King 2013). For example, in a group of brown capuchins, a male repeatedly lifted/pulled the head and tail of a dead adult female and dragged the corpse (De Marco et al. 2020).

The post-mortem cannibalism phase began 12 min after the mother first actively discarded the corpse (Video 7). It is difficult to say if the discarding was intentional or not; however, until then the mother had never separated from the corpse, and immediately after discarding it both the mother and an adult male alternated in dragging it around. Regardless of its underlying motivation, this behavior appeared to signal a shift in the mother’s attitude towards her baby, including a significant reduction in grooming and inspection of the corpse (Fig. 1), in a transition from a proactive (e.g., grooming and inspecting) to a defensive (e.g., keeping it close, avoiding others’ interactions) behavior towards it, notably in response to attempts by other group members to steal it.

Although we cannot exclude a role of captivity in the emergence of cannibalism, in the days before the infant’s death, we observed no increase in aggression directed to the mother or the infant by other group members; instead, only affiliative behaviors were seen. Moreover, several episodes of cannibalism of dead infants have reported in wild primates (e.g., tamarins, Culot et al. 2011; orangutans, Dellatore et al. 2009; Taihangshan macaques, Tian et al. 2016), including filial cannibalism in the two *Pan* species (chimpanzees, Fedurek et al. 2020, bonobos, Tokuyama et al. 2017). As in our case, researchers did not know if the baby died as a result of infanticide. In bonobos and chimpanzees, the mothers consumed parts of the corpse and shared meat with other group members. The rare cases of maternal cannibalism observed in monkeys (Culot et al. 2011; De Marco et al. 2018; Tian et al. 2016) and apes usually involve already-dead infants (Dellatore et al. 2009; Fowler and Hohmann 2010). Cannibalism may appear an adaptive evolutionary trait if we consider the high reproductive energy investment of primate mothers. Recovering energy after gestation can improve the mother’s reproductive success. The absence of sharing the carcass with other group members by the drill mother supports the hypothesis of the nutritional benefit of cannibalism (Bose 2022).

Other factors that should be considered as possible influences on maternal cannibalism include the health state of the newborn. For example, in one case in wild bonobos the infant was probably stillborn (Tokuyama et al. 2017). A second potential factor is the baby’s age. The younger the baby, the less likely the mother–infant attachment has become sufficiently strong to preclude maternal cannibalism in the event of infant death. Notable in the case reported here the baby was not in good health at birth, it died after few days, and it was consumed exclusively by the mother. Although we cannot draw any firm conclusions about motivation or potential benefits of the mother’s behaviors, publication of these types of events are important for both quantitative and qualitative evaluations of the wide range of reported post-mortem behaviors (Nakagawa 2021; Freire Filho et al. 2022).

## Supplementary Information

Below is the link to the electronic supplementary material.

Supplementary file1 (DOCX 16 KB)

Supplementary file2 (XLSX 10 KB)

Supplementary file3 (MP4 8656 KB)

Supplementary file4 (MP4 9288 KB)

Supplementary file5 (MP4 12524 KB)

Supplementary file6 (MP4 15420 KB)

Supplementary file7 (MP4 25099 KB)

Supplementary file8 (MP4 11062 KB)

Supplementary file9 (MP4 12549 KB)

Supplementary file10 (MP4 4508 KB)

Supplementary file11 (MP4 8035 KB)

Supplementary file12 (MP4 6268 KB)

## Data Availability

The raw data are provided in the Supplementary Material section.
