# Supplementary material for: Record of thanatology and cannibalism in drills (Mandrillus leucophaeus)
Source: Primates. 2023 Jun 27;64(5):475–81. doi: 10.1007/s10329-023-01075-8 (PMC10474171; doi:10.1007/s10329-023-01075-8)
Supplement: Supplementary file 1 — Supplementary file1 (DOCX 16 KB) [file 10329_2023_1075_MOESM1_ESM.docx]

**Table S1: Definitions of the behavioral patterns observed in the group of drills under study**

| Category | Pattern | Definition |
| --- | --- | --- |
| PROXIMITY | approaching | One animal moves towards another subject |
|  | following | One animal walks right behind another subject on the same route |
|  | moon walking | One animal walks backwards |
|  | present rear | One animal gets closer to another subject and expose the rear towards it |
|  | proximity | Standing, sitting, resting within 2.5 m from another subject |
|  | touch attempt | One animal tries to touch with its hand another subject |
| CONTACT | body contact | One animal stays in physical contact with another subject |
|  | touching | One subject touches another animal (not any specific part of the body) |
|  | holding | One animal grasps someone or something in its hands |
|  | muzzle contact | One animal rubs the muzzle on another subject |
|  | nose contact | One animal touches the body of another subject with its nose |
|  | nose-to-nose contact | Two subjects touch their nose reciprocally |
|  | play | One animal engages in solitary playful (e.g., object play, play jump) or social playful patterns (e.g., chasing, pushing, slapping, biting, play faces) |
|  | touching rear | One animal directs its genital region towards another subject who touches it |
|  | ventral carrying | One animal carries on the ventral side of its body another subject |
| INSPECT | face-to-face | Two subjects engage in a mutual gazing interaction |
|  | gaze engagement | One subject tries to engage the gaze of another subject, while lowering their forelimbs (elbows usually bent) and rotating its body and head |
|  | inspecting | One animal engages in gentle physical contact, poking and sniffing another subject |
|  | sniffing/hand sniffing/sniffing rear | One animal investigates the environment or another subject by sniffing |
| AGGRESSION | aggressive contact | Biting, attempt to slap, slap, dismiss, push |
|  | avoid/walk away | One animal walks or runs away from another subject |
|  | chase | One animal aggressive chases another subject |
|  | fleeing | One animal runs away from another subject trying to avoid the aggressive interaction |
|  | mount | One animal places its hand on the back of another subject, engaging in repeated thrusts |
|  | staring | A fixed look at another individual |
|  | threatening approach | One animal walks towards another individual while aggressively staring at him |
| EMBRACE |  | One individual puts one or both the arms around another subject |
| GROOMING | grooming/genital grooming | One animal uses the hands to clean the fur of another subject, occasionally using tongue, lips or teeth |
| SELF-DIRECTED | self-grooming | One animal uses its hands picking through its own fur |
|  | body-head shaking | One animal moves its body or head by rotating them repeatedly |
|  | self-scratching | One animal scratches any part of the body (e.g. head) using its fingers |
| REARING | breast feeding | One animal (in particular, the mother) holds the baby on her chest while breast feeding it |
